# Primary endometrioid carcinoma of the uterosacral ligament arising from deep infiltrating endometriosis 6 years after bilateral salpingo-oophorectomy due to atypical proliferative endometrioid tumor of the ovary: a rare case report

**DOI:** 10.1186/s12957-020-02105-1

**Published:** 2020-12-11

**Authors:** Yoshiaki Ota, Kuniaki Ota, Toshifumi Takahashi, Soichiro Suzki, Rikiya Sano, Ikuko Ota, Takuya Moriya, Mitsuru Shiota

**Affiliations:** 1 Department of Gynecological Oncology, Kawasaki Medical School, Kurashiki, Japan; 2 Fukushima Medical Center for Children and Women, Fukushima Medical University, 1 Hikarigaoka, Fukushima City, Fukushima 960-1295 Japan; 3 Department of Gynecology, Kurashiki Heisei Hospital, Kurashiki, Japan; 4 Department of Pathology, Kawasaki Medical School, Kurashiki, Japan

**Keywords:** Endometriosis, Deep infiltrating endometriosis, Malignant transformation, Metachronous cancer

## Abstract

**Background:**

Endometriosis can potentially lead to the development of a malignant tumor. Most malignant tumors arising from the endometriosis originate from the ovarian endometrioma, whereas those arising from extragonadal lesions are rare. We report a rare case of endometrioid carcinoma that developed from deep infiltrating endometriosis in the uterosacral ligament 6 years after treatment for atypical proliferative endometrioid tumor of the ovary in a 48-year-old woman.

**Case presentation:**

Six years ago, the patient underwent laparoscopic bilateral salpingo-oophorectomy for her right ovarian tumor with atypical proliferative (borderline) endometrioid tumor accompanied by ovarian endometrioma. The solid tumor in the cul-de-sac was detected during follow-up using magnetic resonance imaging. Positron emission tomography/computed tomography revealed an abnormal accumulation of ^18^F-fluorodeoxyglucose at the tumor site. Thus, tumor recurrence with borderline malignancy was suspected. The patient underwent diagnostic laparoscopy followed by hysterectomy and partial omentectomy. Retroperitoneal pelvic lymphadenectomy and para-aortic lymphadenectomy were also performed. The cul-de-sac tumor at the left uterosacral ligament was microscopically diagnosed as invasive endometrioid carcinoma arising from deep infiltrating endometriosis. The final diagnosis was primary stage IIB peritoneal carcinoma. The patient received six courses of monthly paclitaxel and carboplatin as adjuvant chemotherapy. The patient showed no evidence of recurrence for 2 years after the treatments.

**Conclusion:**

This study reports a rare case of metachronous endometriosis-related malignancy that developed 6 years after treatment for borderline ovarian tumor. If endometriosis lesions remain after bilateral salpingo-oophorectomy, the physician should keep the malignant nature of endometriosis in mind.

## Background

Endometriosis is a common benign gynecological disorder associated with pelvic pain and/or infertility [1, 2]. It is characterized by the development of uterine glandular and stromal endometrium-like tissues at ectopic locations such as the peritoneum, ovaries, and other distant extra-pelvic lesions [2]. Deep infiltrating endometriosis (DIE) is a subtype wherein the pathological tissue can penetrate more than 5 mm beneath the peritoneum [3]. The incidence of DIE among all endometriosis cases is 20%, and the lesions are most frequently located in the uterosacral ligaments [4].

Although most endometrioses are benign, approximately 0.5–1% of cases develop a malignancy [5]. Most malignant tumors arising from endometriosis are derived from the ovarian endometrioma. However, malignant tumors arising from superficial peritoneal endometriosis lesions or DIEs are rare and standard therapy has not yet been established [6–9]. Hence, endometriosis can be malignant not only in the ovarian endometrioma but also in extra-gonadal endometriosis; nevertheless, there have been few reports of malignant tumors occurring as metachronous tumors in these endometriosis lesions [10]. Here, we report a rare case of endometrioid carcinoma arising from DIE in the uterosacral ligament 6 years after bilateral salpingo-oophorectomy due to the occurrence of an atypical proliferative endometrioid tumor of the ovary.

## Case presentation

**Fig. 1:**
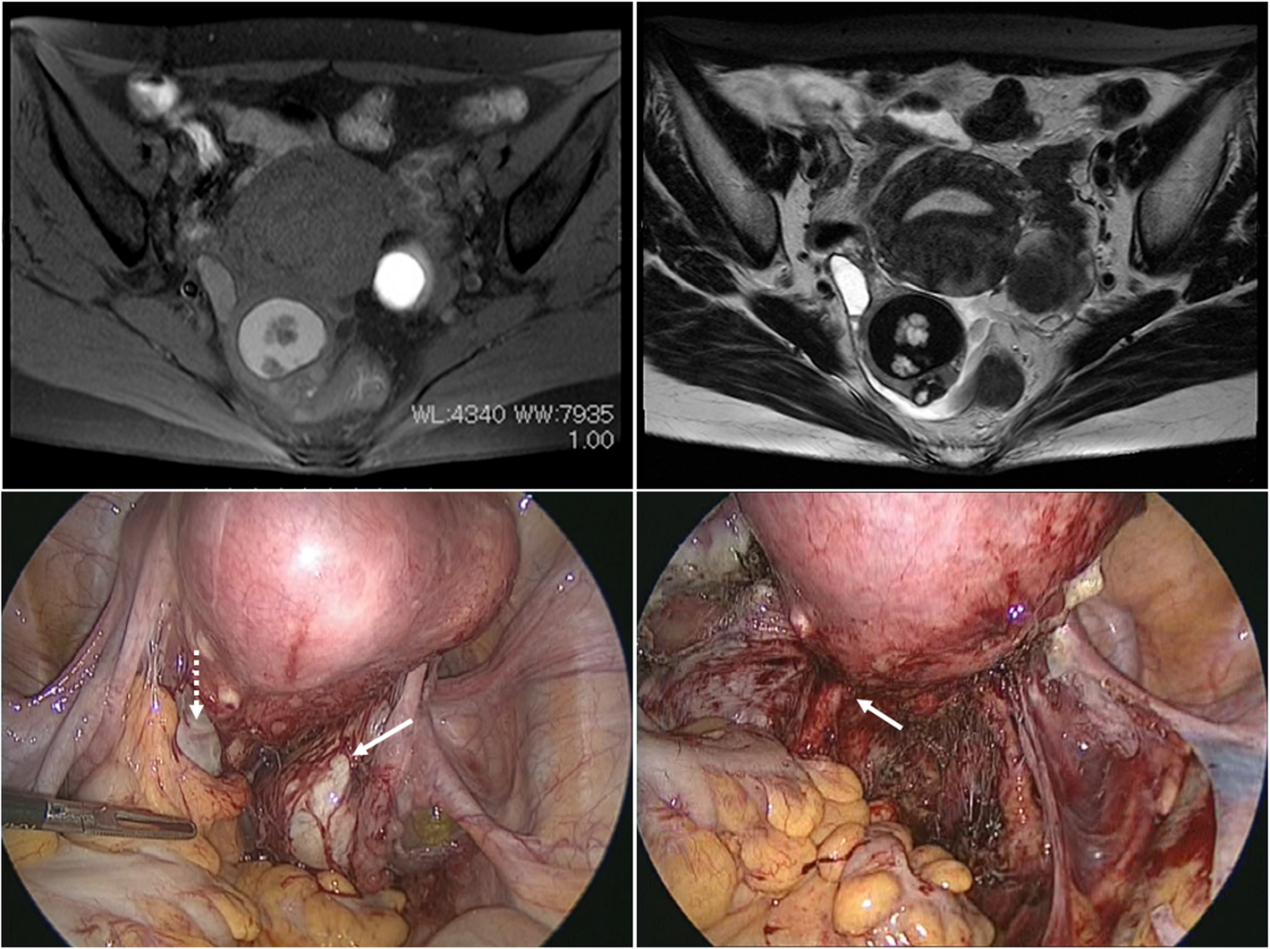
Magnetic resonance images show a right ovarian tumor with a mural nodule and a left ovarian endometrioma. **a** Transverse T1-weighted fat-saturated image after gadolinium enhancement. **b** Transverse T2-weighted image. **c** Laparoscopic findings before surgery of the bilateral ovarian mass with adnexal adhesion. White dotted arrow indicates left ovarian endometrioma, and white solid arrow indicates right ovarian tumor. **d** Laparoscopic findings after bilateral salpingo-oophorectomy. White arrow indicates lesions suspected as deep infiltrating endometriosis lesions in the left uterosacral ligament

A 48-year-old gravida 2, para 2 (vaginal delivery) Japanese woman presented with a history of severe pelvic pain. She was 13 years old at menarche, had a regular menstrual cycle, and presented with dysmenorrhea. She had no previous history of hormone therapy, including oral contraceptives. She had undergone laparoscopic bilateral salpingo-oophorectomy (BSO) when she was 42 years old for her right- and left-sided ovarian tumors. Magnetic resonance imaging (MRI) revealed a 4.4-cm-diameter solid and cystic ovarian tumor of the right ovary with significant gadolinium enhancement and a 3.6-cm-diameter endometrial cyst of the left ovary (Fig. 1a, b). Levels of the tumor markers, carbohydrate antigen 19-9 (CA19-9) and CA-125, were 57 U/ml (reference < 37.0 U/mL) and 66 U/ml (reference < 35.0 U/mL), respectively. Laparoscopic findings showed the presence of bilateral ovarian tumors with severe adhesions around the cul-de-sac due to the development of endometriosis (Fig. 1c). After laparoscopic BSO, a residual endometriotic lesion suspected to be a DIE lesion was detected in the left uterosacral ligament (Fig. 1d).

**Fig. 2:**
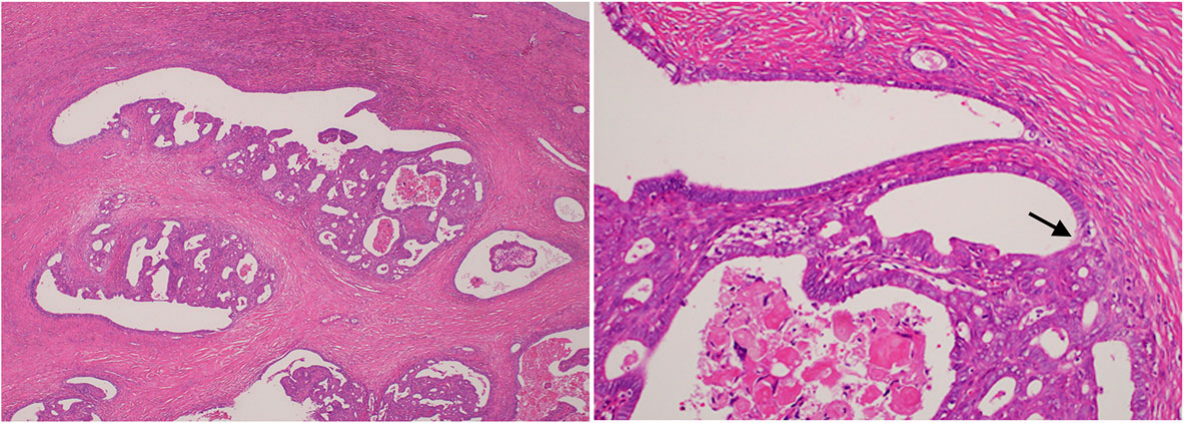
Histopathological findings on the right ovarian tumor during initial surgery. **a** Photograph of microscopic findings of the right ovarian tumor with low magnification. **b** Photograph of microscopic findings of the right ovarian tumor with high magnification. The black arrow indicates a transition from an endometriotic tissue to a cancerous lesion (front formation)

The pathological diagnosis of the right and left ovaries was atypical proliferative endometrioid tumor and ovarian endometrioma, respectively. Histopathological findings of the right ovarian tumor showed complex glandular proliferation with stratified, enlarged nuclei and clear chromatin surrounded by fibrocollagenous stroma. Stromal invasion was not evident (Fig. 2a). A direct transition from a non-atypical endometriotic gland to an atypically proliferative lesion was observed (Fig. 2b). The tumor was limited to the right ovary, and the capsule was intact.

**Fig. 3:**
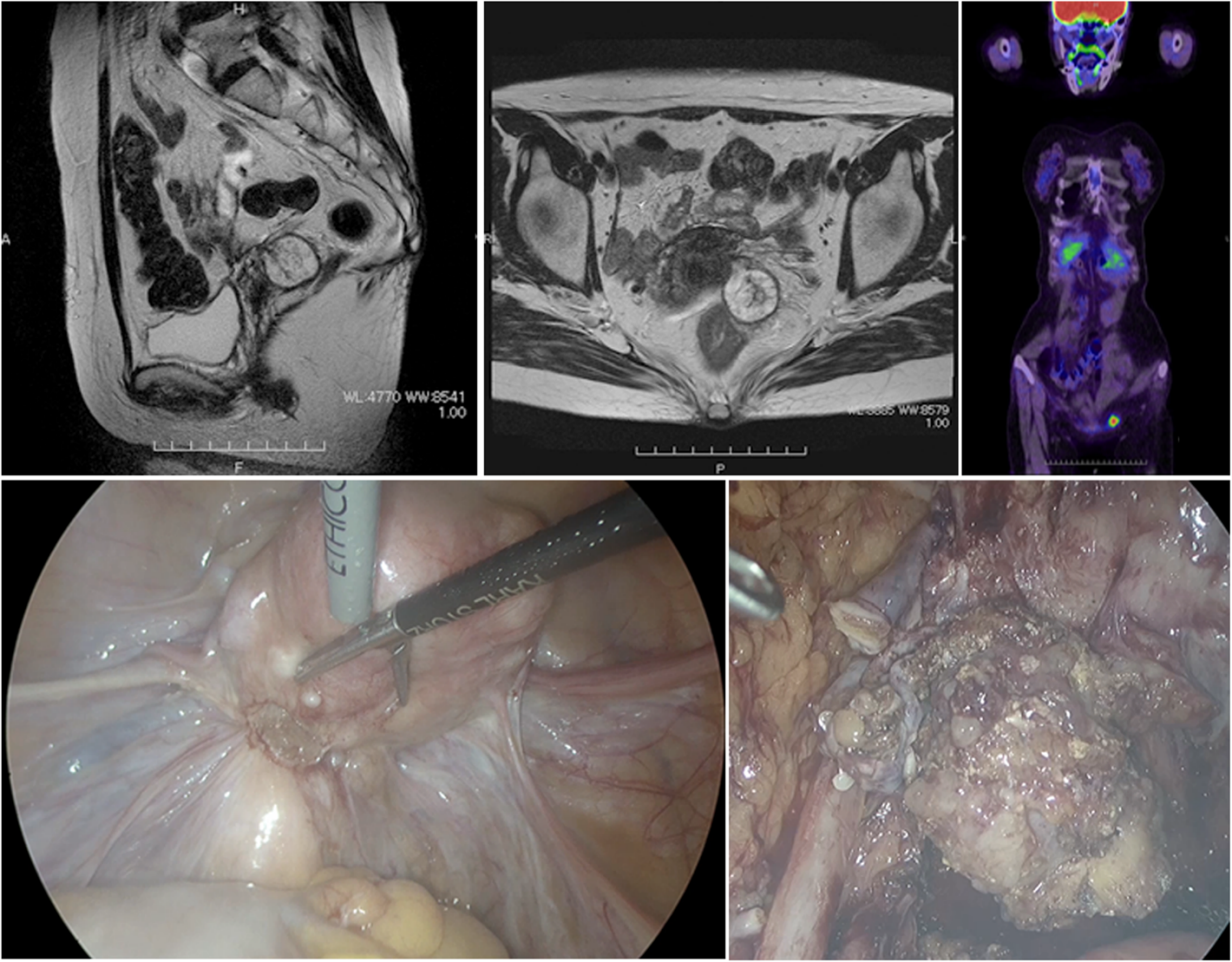
Magnetic resonance images and laparoscopic findings on the tumor located in the cul-de-sac. **a** Sagittal T2-weighted image. **b** Transverse T2-weighted image. **c** Whole-body ^18^F-fluorodeoxyglucose (FDG) positron emission tomography (PET) scan image. FDG-PET scan shows high FDG uptake at the left side of the pelvic cavity. **d** Photograph of laparoscopic findings of the cul-de-sac tumor in the left uterosacral ligament. **e** Photograph of the tumor excavated from the cul-de-sac

With the right ovarian tumor being diagnosed as a borderline malignancy, we suggested a staging laparotomy with definitive completion surgery to the patient, as she did not desire to retain childbearing function. However, the patient declined, and she was instead carefully followed up quarterly. Contrast-enhanced computed tomography (CT) was performed every 6 months during the 5-year postoperative follow-up period, with no recurrence of disease. Six years after the operation, ascites was identified in the pelvic cavity along with a solid tumor, localized to the left side of the cul-de-sac. There was no lymph node swelling or peritoneal dissemination (Fig. 3a, b). She underwent an 18F-fluorodeoxyglucose (FDG) positron emission tomography/CT (PET/CT) examination. The PET/CT revealed an abnormal accumulation of FDG at the tumor site (Fig. 3c). The levels of tumor markers CA19-9 and CA-125 were 81 U/ml (reference < 37.0 U/mL) and 45 U/ml (reference < 35.0 U/mL), respectively. Therefore, recurrent ovarian tumor with borderline malignancy was suspected. To assess the feasibility of cytoreduction, diagnostic laparoscopy was performed. Laparoscopic findings showed that the tumor in the cul-de-sac was buried and firmly attached to the sigmoid colon with no apparent ascites or peritoneal dissemination (Fig. 3d). A peritoneal lavage cytology was performed. The tumor was found at the site of the DIE lesions that were present 6 years before the initial laparoscopic surgery. The tumor buried in the cul-de-sac was removed through laparoscopic surgery (Fig. 3e).

**Fig. 4:**
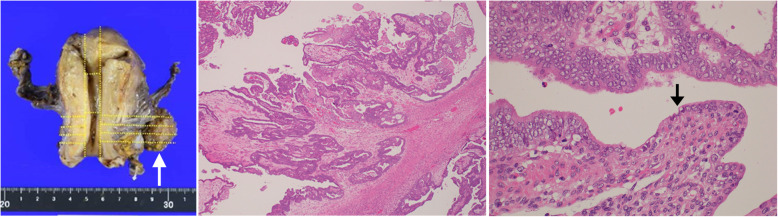
Histopathological findings on the cul-de-sac tumor. **a** Photograph of macroscopic findings of the extirpated cul-de-sac tumor attached to the uterus. White arrow indicates the extirpated cul-de-sac tumor. **b** Photograph of microscopic findings of the cul-de-sac tumor with low magnification. **c** Photograph of microscopic findings of the cul-de-sac tumor with high magnification. Black arrow indicates a transition from the lesion of endometriosis to the lesion of endometrial carcinoma (front formation)

We also performed a hysterectomy, partial omentectomy, and retroperitoneal pelvic and para-aortic lymphadenectomy by laparotomy. Macroscopically, the tumor was localized in the left parametrium (Fig. 4a). Histologically, it was a well-differentiated endometrioid carcinoma with a papillary-cribriform structure, in which cancerous glands with stratified and enlarged nuclei and stromal invasion were found (Fig. 4b). At the edge of the tumor, an ectopic endometrial gland of the DIE was directly connected to the cancerous glands (Fig. 4c). Therefore, a pathological diagnosis of endometrioid carcinoma arising from the DIE lesions was made. The peritoneal lavage cytology was negative, and no lymph node metastases were identified. The final diagnosis was a primary peritoneal carcinoma of stage IIB based on the International Federation of Gynecology and Obstetrics staging system. The patient received six courses of monthly paclitaxel and carboplatin as adjuvant chemotherapy for her peritoneal cancer. There was no evidence of recurrence for 2 years after treatment. The patient has provided informed consent for publication of the case.

## Discussion

We report a case of endometrioid carcinoma arising from DIE lesions in the uterosacral ligament 6 years after treatment of borderline ovarian malignancy. This is a rare case of metachronous endometriosis-related carcinoma.

A malignant transformation is rare in endometriosis, occurring in only approximately 1% of cases [5]. There are several reports regarding the incidence of cancer development from ovarian endometriosis. A prospective cohort analysis showed that the incidence of ovarian cancer was 0.7% (46/6398) in women with endometrioma [11]. The frequency of malignant transformation of the endometriosis in extra-ovarian sites, such as the peritoneal and other extra-pelvic cavities, was reported as 0.2% [12].

The preferred sites of DIE in the pelvis are the uterosacral ligament and rectovaginal septum, and there are several reports of malignancies arising from these areas. Recently, Yang et al. reported and reviewed the 10 cases of malignant tumors located in the rectovaginal septum related to endometriosis [13]. Although even more rare, Tarumi et al. reported and reviewed 9 cases of malignant transformation arising from DIE in the bladder [14]. The histological type of the malignancy that occurred was endometrioid and clear cell adenocarcinoma in both the rectovaginal septum and the bladder.

Conversely, the histological types for epithelial ovarian cancer related to ovarian endometrioma include the endometrioid, clear cell, and serous types. Pearce et al. investigated the relationship between endometriosis and various histological types of ovarian cancer, including borderline malignancy of ovarian tumors; however, no association was observed between endometriosis and the risk of mucinous or high-grade serous carcinoma or the histological type of borderline malignancy [15].

Endometriosis with certain molecular genetic features may have a potential for carcinogenesis [16]. Some reports described mutations of the *ARID1A* gene in 30% of women with endometrioid carcinoma derived from ovarian endometrioma [17, 18]. Other mutations, such as those of the *CTNNB1* and *PTEN* genes, have also been reported in endometrioid carcinoma related to endometriosis [19]. Most of the studies regarding genetic abnormalities in women with endometriosis were analyses of women with ovarian endometrioma.

Genetic factors may also be involved in cases of ovarian cancer derived from peritoneal lesions of the endometriosis or DIE lesions. Recently, Anglesio et al. reported that DIE without cancer was associated with some somatic mutations, including those of the *ARID1A*, *PIK3CA*, *KRAS*, or *PPP2R1A* genes, all of which are cancer driver mutations [20]. These mutations may lead to extra-ovarian endometriosis-related carcinomas, such as those from the vagina, fallopian tube or mesosalpinx, pelvic sidewall, colon, or parametrium [9]. Here, the patient might have some somatic mutations in the cancer lesions. Since the carcinogenesis of ovarian endometriosis and peritoneal endometriosis may differ, somatic mutations in the initial borderline malignant ovarian tumor and ovarian cancer arising from DIE may also vary.

In this study, the patient had metachronous malignancies derived from endometriotic lesions. Uehara et al. reported the first case similar to ours, and it was a case of metachronous cancer of two different histological subtypes of endometriosis-related tumors [10]. In their report, the second primary cancer was peritoneal seromucinous carcinoma associated with peritoneal endometriosis after treatment of ovarian clear cell carcinoma with endometriosis. In the present case, the borderline malignancy in the right ovary was considered to originate from the malignant transformation of the endometriosis, evidenced by the presence of front formation, which is a transition from endometriosis to atypical proliferation with nuclear atypia but without stromal invasion. Most histological types of epithelial borderline ovarian tumors are serous and mucinous, and endometrioid types are rare [21]. Atypical proliferative endometrioid ovarian tumor account for a minuscule 0.2% of all ovarian epithelial tumors [21]. Although this type of tumor is rare, most of them have been reported as stage 1, and with a good prognosis [22]. The histopathological findings of the tumor found 6 years after the initial surgery was an invasive endometrioid adenocarcinoma and the tumor was considered to originate from the DIE lesions due to the presence of front formation. Initially, the lesion was suspected to be a recurrent lesion of the right borderline ovarian tumor. However, laparoscopy revealed that the lesion was at the site of the uterosacral ligament on the left side from the time of surgery 6 years earlier. Although some problems were encountered, such as the lack of accurate surgical staging at the time of initial surgery, it is reasonable to assume that these metachronous endometriosis-related malignancies have a different origin, considering the good prognosis of atypical proliferative endometrioid ovarian tumors.

Although there appears to be an association between ovarian endometrioma and epithelial ovarian cancer, endometriosis is not considered a premalignant lesion, and screening is not recommended. In general, the guideline from the European Society of Human Reproduction and Embryology does not support surgical resection of asymptomatic lesions of peritoneal endometriosis [23]. To date, there are no data indicating that prophylactic removal of ovarian endometrioma or peritoneal lesions of endometriosis or DIE lesions can reduce the risk of epithelial ovarian cancer.

In conclusion, we reported a rare case of metachronous endometriosis-related malignancies, particularly a primary peritoneal endometrioid carcinoma arising from DIE lesions 6 years after the occurrence of borderline ovarian tumors. As endometriosis has a potential for malignant transformation, long-term follow-up is necessary for survivors of ovarian malignancy with endometriosis.

## Data Availability

Not applicable.

## Abbreviations

DIE: Deep infiltrating endometriosis
BSO: Bilateral salpingo-oophorectomy
MRI: Magnetic resonance imaging
CA19-9: Carbohydrate antigen 19-9
FDG: 18F-fluorodeoxyglucose
PET/CT: Positron emission tomography/computed tomography

## References

[1] de Ziegler D, Borghese B, Chapron C (2010). Endometriosis and infertility: pathophysiology and management. Lancet..

[2] Giudice LC (2010). Clinical practice. Endometriosis N Engl J Med..

[3] Vercellini P, Frontino G, Pietropaolo G, Gattei U, Daguati R, Crosignani PG (2004). Deep endometriosis: definition, pathogenesis, and clinical management. J Am Assoc Gynecol Laparosc..

[4] Fauconnier A, Chapron C, Dubuisson JB, Vieira M, Dousset B, Breart G (2002). Relation between pain symptoms and the anatomic location of deep infiltrating endometriosis. Fertil Steril..

[5] Wei JJ, William J, Bulun S (2011). Endometriosis and ovarian cancer: a review of clinical, pathologic, and molecular aspects. Int J Gynecol Pathol..

[6] Seki K, Ishikawa H, Hashimoto R, Mitsuhashi A, Ikeda J-I, Shozu M (2020). Development of localized cul-de-sac endometrioid carcinoma associated with deep infiltrating endometriosis during remission of early endometrial cancer. Gynecol Oncol Rep.

[7] Wu WC, Hsiao MW, Ye JC, Hung YC, Chang WC (2009). Malignant transformation of extragonadal endometriosis: a case report. Eur J Gynaecol Oncol..

[8] Marchand E, Hequet D, Thoury A, Barranger E. Malignant transformation of superficial peritoneal endometriosis lesion. BMJ Case Rep. 2013;2013:bcr2012007730. 10.1136/bcr-2012-007730 PMC3762544 23978494

[9] Leiserowitz GS, Gumbs JL, Oi R, Dalrymple JL, Smith LH, Ryu J, Scudder S, Russell AH (2003). Endometriosis-related malignancies. Int J Gynecol Cancer..

[10] Uehara T, Yoshida H, Tate K, Kato T (2019). Metachronous occurrence of two different histological subtypes of endometriosis-related neoplasms. Gynecol Oncol Rep..

[11] Kobayashi H, Sumimoto K, Moniwa N, Imai M, Takakura K, Kuromaki T, Morioka E, Arisawa K, Terao T (2007). Risk of developing ovarian cancer among women with ovarian endometrioma: a cohort study in Shizuoka, Japan. Int J Gynecol Cancer..

[12] Benoit L, Arnould L, Cheynel N, Diane B, Causeret S, Machado A, Collin F, Fraisse J, Cuisenier J (2006). Malignant extraovarian endometriosis: a review. Eur J Surg Oncol..

[13] Yang H, Gu JJ, Qi Y, Zhao W, Wang XL (2019). Endometrioid adenocarcinoma of the rectovaginal septum with invasion of the rectum: a case report and review of literature. World J Surg Oncol..

[14] Tarumi Y, Mori T, Kusuki I, Ito F, Kitawaki J (2015). Endometrioid adenocarcinoma arising from deep infiltrating endometriosis involving the bladder: a case report and review of the literature. Gynecol Oncol Rep..

[15] Pearce CL, Templeman C, Rossing MA, Lee A, Near AM, Webb PM, Nagle CM, Doherty JA, Cushing-Haugen KL, Wicklund KG (2012). Association between endometriosis and risk of histological subtypes of ovarian cancer: a pooled analysis of case-control studies. Lancet Oncol..

[16] Kurman RJ, Shih I-M (2016). The dualistic model of ovarian carcinogenesis: revisited, revised, and expanded. Am J Pathol..

[17] Wiegand KC, Shah SP, Al-Agha OM, Zhao Y, Tse K, Zeng T, Senz J, McConechy MK, Anglesio MS, Kalloger SE (2010). ARID1A mutations in endometriosis-associated ovarian carcinomas. N Engl J Med..

[18] Ayhan A, Mao TL, Seckin T, Wu CH, Guan B, Ogawa H, Futagami M, Mizukami H, Yokoyama Y, Kurman RJ (2012). Loss of ARID1A expression is an early molecular event in tumor progression from ovarian endometriotic cyst to clear cell and endometrioid carcinoma. Int J Gynecol Cancer..

[19] McConechy MK, Ding J, Senz J, Yang W, Melnyk N, Tone AA, Prentice LM, Wiegand KC, McAlpine JN, Shah SP (2014). Ovarian and endometrial endometrioid carcinomas have distinct CTNNB1 and PTEN mutation profiles. Mod Pathol..

[20] Anglesio MS, Papadopoulos N, Ayhan A, Nazeran TM, Noe M, Horlings HM, Lum A, Jones S, Senz J, Seckin T (2017). Cancer-associated mutations in endometriosis without cancer. N Engl J Med..

[21] Roth LM, Emerson RE, Ulbright TM (2003). Ovarian endometrioid tumors of low malignant potential: a clinicopathologic study of 30 cases with comparison to well-differentiated endometrioid adenocarcinoma. Am J Surg Pathol..

[22] Bell KA, Kurman RJ (2000). A clinicopathologic analysis of atypical proliferative (borderline) tumors and well-differentiated endometrioid adenocarcinomas of the ovary. Am J Surg Pathol..

[23] Dunselman GAJ, Vermeulen N, Becker C, Calhaz-Jorge C, D'Hooghe T, De Bie B, Heikinheimo O, Horne AW, Kiesel L, Nap A (2014). ESHRE guideline: management of women with endometriosis †. Hum Reprod..

